# 伏美替尼敏感的*EGFR* 20号外显子H773_V774delinsLM突变肺腺癌1例

**DOI:** 10.3779/j.issn.1009-3419.2025.102.19

**Published:** 2025-06-30

**Authors:** Rongzhen LI, Yan XU, Xiaoxing GAO, Minjiang CHEN, Wei ZHONG, Mengzhao WANG

**Affiliations:** 100730 北京，中国医学科学院，北京协和医学院，北京协和医院呼吸与危重症医学科; Department of Pulmonary and Critical Care Medicine, Peking Union Medical College Hospital, Chinese Academy of Medical Sciences and Peking Union Medical College, Beijing 100730, China

**Keywords:** 肺肿瘤, *EGFR*突变, 罕见突变, 伏美替尼, Lung neoplasms, *EGFR* mutations, Rare mutations, Furmonertinib

## Abstract

表皮生长因子受体（epidermal growth factor receptor, *EGFR*）20号外显子突变是非小细胞肺癌中的罕见突变，其中H773_V774delinsLM复杂突变尤为罕见，仅占所有*EGFR*突变的0.2%-1%。*EGFR*罕见突变通常被认为对第一、二代*EGFR*-酪氨酸激酶抑制剂（*EGFR*-tyrosine kinase inhibitors, EGFR-TKIs）耐药，尽管第三代EGFR-TKIs在部分罕见突变中显示出一定疗效，但针对H773_V774delinsLM突变患者的治疗数据仍十分有限，其疗效和安全性尚未明确。本文报告了首例经伏美替尼治疗后获得显著肿瘤消退的*EGFR* H773_V774delinsLM突变肺腺癌，以期为临床中这类罕见突变患者可能的治疗方案提供新的见解。

## Abstract (en)

Epidermal growth factor receptor (*EGFR*) exon 20 mutations represent a rare subset of genetic alterations in non-small cell lung cancer (NSCLC). Among them, the complex mutation H773_V774delinsLM is exceedingly uncommon, accounting for only 0.2%-1% of all *EGFR* mutations. It is currently believed that rare *EGFR* mutations are generally resistant to the first- and second-generation *EGFR*-tyrosine kinase inhibitors (EGFR-TKIs). Although the third-generation EGFR-TKIs have shown some efficacy in certain rare mutations, clinical evidence regarding their use in NSCLC patients with the H773_V774delinsLM mutation remains sparse, and their efficacy and safety are yet to be clarified. Here, we present the first documented case of a patient with *EGFR* H773_V774delinsLM-mutant lung adenocarcinoma who experienced remarkable tumor regression following treatment with furmonertinib. This case highlights the potential utility of furmonertinib in treating patients with this rare *EGFR* mutation and may provide valuable insight into emerging treatment strategies for similarly affected patients.

表皮生长因子受体（epidermal growth factor receptor, *EGFR*）突变是非小细胞肺癌（non-small cell lung cancer, NSCLC）中最常见的驱动基因突变^[1]^。近年来，*EGFR*-酪氨酸激酶抑制剂（*EGFR*-tyrosine kinase inhibitors, EGFR-TKIs）的应用显著改善了经典*EGFR*敏感突变（包括19号外显子缺失突变和21号外显子L858R突变）患者的预后。然而，EGFR-TKIs在*EGFR*罕见突变患者中的治疗可行性尚不明确，并且不同EGFR-TKIs的疗效存在异质性^[2]^。在*EGFR*罕见突变中，20号外显子插入（exon 20 insertion, ex20ins）突变最为常见，占所有*EGFR*突变的1%-9%^[3-5]^。这些插入突变约10%位于*EGFR*酪氨酸激酶结构域αC-螺旋的C末端（氨基酸761-766），90%位于αC-螺旋后的环（氨基酸767-775）。这些结构变化导致αC-螺旋和磷酸结合环向药物结合口袋移动，形成明显的空间位阻，从而降低了传统EGFR-TKIs的有效性^[3,6,7]^。

除此之外，20号外显子还存在多种点突变，其中H773_V774delinsLM复杂突变尤为罕见，仅占*EGFR*突变的0.2%-1%^[8,9]^，目前关于EGFR-TKIs治疗该突变患者疗效及安全性的临床数据仍十分有限。本文报告了首例经伏美替尼治疗后获得显著肿瘤消退的*EGFR* H773_V774delinsLM突变肺腺癌，以期为临床中这类罕见突变患者可能的治疗方案提供新的见解。

## 1 病例资料

患者男性，43岁，吸烟史10年。2023年12月患者因间断咳嗽就诊，查胸部计算机断层扫描（computed tomography, CT）：右肺上叶团块状软组织密度灶，大小约77 mm×50 mm；两肺门及纵隔多发肿大淋巴结；双肺多发结节。2024年1月5日行支气管镜下右上叶支气管开口处黏膜活检及右肺上叶肿物穿刺活检，病理：肺腺癌，免疫组化：间变性淋巴瘤激酶（anaplastic lymphoma kinase, ALK）（-），细胞角蛋白7（cytokeratin 7, CK7）（+），P40（-），甲状腺转录因子1（thyroid transcription factor 1, TTF-1）（+）。组织样本下一代测序（next-generation sequencing, NGS）基因检测：*EGFR* 20号外显子缺失插入突变c.2318_2320delinsTAA（p.H773_V774delinsLM）；程序性细胞死亡配体1（programmed cell death ligand 1, PD-L1）表达未测。2024年1月10日完善全身正电子发射计算机断层显像（positron emission tomography/CT, PET/CT）：右肺上叶团块，大小约84 mm×53 mm，放射性摄取不均匀异常增高，SUVmax为8.4，考虑恶性病变；两肺门及纵隔多发代谢增高淋巴结，转移可能；双肺多发代谢增高结节，考虑多发转移；余未见明显转移（图1A、1B）。考虑右肺腺癌诊断明确，临床分期cT4N3M1a，IVA期（国际抗癌联盟第八版）。

**图1:**
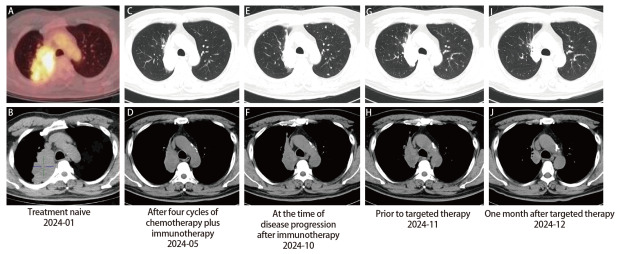
患者病程中胸部影像学检查。 A、B：治疗前（A：PET/CT，B：胸部CT纵隔窗）；C、D：化疗联合免疫治疗4个周期后（C：肺窗，D：纵隔窗）；E、F：免疫治疗后进展时（E：肺窗，F：纵隔窗）；G、H：靶向治疗前（G：肺窗，H：纵隔窗）；I、J：靶向治疗1个月后（I：肺窗，J：纵隔窗）。

由于*EGFR* 20号外显子缺失插入突变并非常见的敏感突变，靶向治疗效果不确定，遂于2024年2月3日起给予4个周期化疗联合免疫治疗，具体为培美曲塞950 mg d1+卡铂500 mg d1+帕博利珠单抗200 mg d1，每21天为1个周期。治疗后患者咳嗽好转，监测血清癌胚抗原（carcinoembryonic antigen, CEA）较治疗前降低（2024-02→2024-05：13.5 ng/mL→8.5 ng/mL），2及4个周期后复查CT评效部分缓解（partial response, PR）（图1C、1D）。后更换为培美曲塞950 mg d1，帕博利珠单抗200 mg d1，每21天为1个周期维持治疗，期间患者一般情况尚稳定，偶有干咳，无头晕、头痛等不适，但监测CEA逐渐升高（2024-05→2024-06→2024-08→2024-09：8.5 ng/mL→13.2 ng/mL→18.9 ng/mL→24.7 ng/mL），6及7个周期后复查CT提示肺部原发灶同前，但双肺结节增多增大（图1E、1F），头磁共振成像（magnetic resonance imaging, MRI）示多发颅内转移（图2A），评效疾病进展（progressive disease, PD），无进展生存期（progression-free survival, PFS）为7个月，重新分期为cT4N3M1c，IVB期。*EGFR* H773_V774delinsLM突变在氨基酸水平为错义突变，针对*EGFR* ex20ins的靶向药物在此类患者中的获益不明确，后患者于2024年10月16日改为多西他赛130 mg d1+帕博利珠单抗200 mg d1治疗，每21天为1个周期，但患者咳嗽逐渐加重，CEA持续升高（2024-09→2024-10→2024-11：24.7 ng/mL→31.1 ng/mL→38.9 ng/mL），2个周期后复查颅内病变继续增大（图2B），肺部病灶同前（图1G、1H），评效疾病稳定（stable disease, SD）。考虑患者携带*EGFR*罕见突变，在患者充分知情同意后，2024年11月29日起予甲磺酸伏美替尼160 mg qd口服靶向治疗。服药后咳嗽好转，1个月后复查CEA明显降低（10.8 ng/mL），胸部CT示右肺上叶肺门旁占位较前缩小（58 mm→32 mm），双肺多发结节较前减少、缩小（图1I、1J），头MRI示颅内多发转移瘤减少、减小（图2C），评效PR。截止目前，患者已口服伏美替尼4个月，CEA降至正常（1.5 ng/mL），肺部病灶大致同前，颅内病灶消失（图2D），评效维持PR，主要不良反应为轻度腹泻。

**图2:**
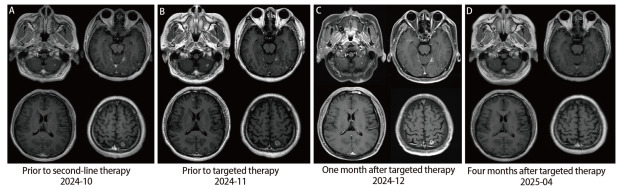
患者病程中头磁共振检查。 A：二线治疗前；B：靶向治疗前；C：靶向治疗1个月后；D：靶向治疗4个月后。

## 2 讨论

目前，已有多种药物对*EGFR* ex20ins突变的NSCLC患者展现出治疗潜力。作为首个获批的靶向*EGFR*/c-MET的双特异性抗体，埃万妥单抗在经含铂化疗后进展的*EGFR* ex20ins突变患者中客观缓解率（objective response rate, ORR）为40%，中位PFS为8.3个月，中位总生存期（overall survival, OS）为22.8个月^[10]^，III期PAPILLON研究^[11]^进一步证实一线埃万妥单抗联合化疗治疗*EGFR* ex20ins NSCLC可显著提升ORR至73%、中位PFS至11.4个月。莫博替尼作为口服TKIs，曾获美国食品药品监督管理局（Food and Drug Administration, FDA）加速批准用于*EGFR* ex20ins突变的晚期NSCLC患者的后线治疗^[12]^，但因III期研究中一线治疗未达优效性终点而被撤回^[13]^。Poziotinib能够克服*EGFR* ex20ins空间位阻而产生不可逆的结合，但疗效受突变位置影响显著（近环突变ORR为46%，远环突变无效）^[7,14]^。新型TKIs药物Zipalertinib在I/II期试验中后线治疗ORR达41%，中位PFS为12个月，且安全性可控，已获FDA突破性疗法认定^[15-17]^。此外，舒沃替尼在中国和国际多中心患者中后线治疗ORR高达60.8%和53.3%^[18-20]^，汇总分析显示舒沃替尼单药一线治疗*EGFR* ex20ins突变患者的ORR达77.8%^[21]^，安全性谱与其他EGFR-TKIs相似，以腹泻和皮疹为主，300 mg剂量下3级及以上治疗相关不良反应发生率为39.2%-45%，其中最常见的是血肌酸磷酸激酶升高（13.5%-17%）和腹泻（7.3%-8%）^[19,22]^，显示出良好的应用前景，目前舒沃替尼针对*EGFR* ex20ins的III期临床试验（WU-KONG28）以及纳入更多突变类型的WU-KONG15试验正在进行中。这些药物给*EGFR* ex20ins突变的NSCLC患者带来了新的治疗选择，但上述研究并未纳入*EGFR* 20号外显子错义突变。*EGFR* 20号外显子H773_V774delinsLM突变在NSCLC中极为罕见，目前文献中仅有6例相关报道^[8,9,23-25]^，其中5例患者接受了EGFR-TKIs治疗：2例患者接受厄洛替尼，最佳疗效分别为PD和SD，PFS分别为0和5个月^[8]^；1例患者对一线阿法替尼治疗表现为原发耐药^[23]^；在Position20试验中，1例患者接受双倍剂量奥希替尼治疗，最佳疗效为PR^[24]^；1例患者使用吉非替尼疗效不佳（最佳疗效为PD），换用奥希替尼后获得了持久的肿瘤控制（最佳疗效为SD，PFS为12个月，OS为15个月）^[25]^。

伏美替尼是一种第三代EGFR-TKIs，目前两项Ib期试验^[26,27]^初步验证了伏美替尼在多种*EGFR*罕见突变NSCLC中的疗效。研究中伏美替尼在携带*EGFR* ex20ins等多种罕见突变的NSCLC患者中展现了较高的治疗反应率和较长的缓解持续时间，并且颅内疗效良好。在一项真实世界的回顾性研究^[28]^中，伏美替尼同样显示出良好的全身及颅内疗效和安全性。治疗剂量选择方面，研究^[27,28]^显示，在*EGFR*罕见突变患者中，相比常规剂量（80 mg qd），双倍剂量（160 mg qd）及三倍剂量（240 mg qd）伏美替尼能够提高ORR，且安全性可接受，为高剂量应用伏美替尼提供了依据。

*EGFR* H773_V774delinsLM突变位于αC-螺旋后的环区，蛋白质结构预测模型显示，H773L和V774M氨基酸改变可能通过引发对αC-螺旋的空间位阻，破坏*EGFR*的非激活构象稳定性，导致其组成性激活^[25]^。与典型的*EGFR* ex20ins不同，*EGFR* H773_V774delinsLM突变并不改变氨基酸序列的整体长度，因此在COSMIC数据库和部分文献中被归类为错义突变^[23,25]^，提示其对EGFR-TKIs反应性可能不同于典型*EGFR* ex20ins。临床前模型药物敏感性测试的结果显示，H773L突变、V774M突变对第一代TKIs不敏感，对第二、三代TKIs相对敏感^[29,30]^，但目前还没有针对H773L+V774M突变的药敏报告。本研究报告了1例携带*EGFR* H773_V774delinsLM突变的晚期NSCLC患者，在接受化疗联合免疫治疗后疾病进展，而后使用高剂量伏美替尼治疗，观察到肺内和颅内病灶显著减小、减少，提示*EGFR* H773_V774delinsLM可能对第三代EGFR-TKIs伏美替尼敏感。此外，本例患者对双倍剂量伏美替尼耐受性良好，仅出现轻度腹泻，这与既往研究^[27]^中报道的安全性特征一致。该结果进一步支持伏美替尼在*EGFR* 20号外显子罕见突变患者中的潜在应用价值。

综上所述，本病例提示部分*EGFR*的罕见突变也可能对第三代EGFR-TKIs敏感。对于*EGFR* 20号外显子H773_V774delinsLM突变NSCLC患者，伏美替尼可能是一种有效的治疗选择。由于目前针对该突变的报道有限，仍需进一步的临床研究来验证伏美替尼的疗效。未来需要更大规模的前瞻性研究和真实世界数据来进一步明确*EGFR* 20号外显子罕见突变患者的最佳治疗策略，以优化此类患者的临床管理。
